# Value assessment of PD-1/PD-L1 inhibitors in the treatment of oesophageal and gastrointestinal cancers

**DOI:** 10.3389/fphar.2023.1106961

**Published:** 2023-04-21

**Authors:** Shun-Long Ou, Jing Luo, Hua Wei, Xiao-Li Qin, Qian Jiang

**Affiliations:** ^1^ Department of Pharmacy, Sichuan Clinical Research Center for Cancer, Sichuan Cancer Hospital and Institute, Sichuan Cancer Center, Affiliated Cancer Hospital of University of Electronic Science and Technology of China, Chengdu, China; ^2^ School of Medicine, University of Electronic Science and Technology of China, Chengdu, China; ^3^ Department of Pharmacy, Chengdu Second People’s Hospital, Chengdu, China; ^4^ Department of Pharmacy, The Third People’s Hospital of Chengdu, Chengdu, China

**Keywords:** PD-1/PD-L1 inhibitors, ESMO-MCBS, ASCO-VF, value, cost

## Abstract

**Background:** Evidence of efficacy and safety of programmed cell death 1 (PD-1) and programmed death ligand-1 (PD-L1) checkpoint inhibitors in oesophageal cancer (EC), gastric cancer (GC) and colorectal cancer (CRC) was inconsistent, obscuring their clinical application and decision-making. The aim of this study was to comprehensively evaluate the value of PD-1/PD-L1 inhibitors in EC, GC and CRC to select valuable PD-1/PD-L1 inhibitors, and to assess the association between the value and cost of PD-1/PD-L1 inhibitors.

**Methods:** A comprehensive search of trials of PD-1/PD-L1 inhibitors in EC, GC and CRC was performed in Chinese and English medical databases with a cut-off date of 1 July 2022. Two authors independently applied the ASCO-VF and ESMO-MCBS to assess the value of PD-1/PD-L1 inhibitors. A receiver operating characteristic (ROC) curve was generated to establish the predictive value of the ASCO-VF score to meet the threshold of the ESMO-MCBS grade. Spearman’s correlation was used to calculate the relationship between the cost and value of drugs.

**Results:** Twenty-three randomized controlled trials were identified: ten (43.48%) in EC, five (21.74%) in CRC, and eight (34.78%) in GC or gastroesophageal junction cancer (GEJC). For advanced diseases, ASCO-VF scores ranged from −12.5 to 69, with a mean score of 26.5 (95% *CI* 18.4–34.6). Six (42.9%) therapeutic regimens met the ESMO-MCBS benefit threshold grade. The area under the ROC curve was 1.0 (*p* = 0.002). ASCO-VF scores and incremental monthly cost were negatively correlated (Spearman’s *ρ* = −0.465, *p* = 0.034). ESMO-MCBS grades and incremental monthly cost were negatively correlated (Spearman’s *ρ* = −0.211, *p* = 0.489).

**Conclusion:** PD-1/PD-L1 inhibitors did not meet valuable threshold in GC/GEJC. Pembrolizumab met valuable threshold in advanced microsatellite instability–high CRC. The value of camrelizumab and toripalimab may be more worth paying in EC.

## Introduction

According to GLOBOCAN data, colon cancer, gastric cancer (GC), rectal cancer and oesophageal cancer (EC) are among the top 10 cancers in terms of incidence, and digestive system cancers have become one of the most serious disease burdens (Sung et al., 2021). In recent years, the use of programmed cell death 1 (PD-1) and programmed death ligand-1 (PD-L1) inhibitors in the treatment of digestive system cancers has been proven to improve the survival of patients and has become an important research topicality (Kang et al., 2017; André et al., 2020; Doki et al., 2022). However, our previous study found that the efficacy and safety of PD-1/PD-L1 inhibitors in EC, GC and colorectal cancer (CRC) were inconsistent (Ou et al., 2022), which extremely confused their clinical application and usefulness in aiding decision-making.

The goal of cancer treatment has changed from the traditional disease-centred strategy to a patient-centred strategy, and we should pay more attention to the comprehensive value (safety, quality of life, affordability, etc.) of the therapeutic regimen in addition to its efficacy. The value of anti-tumor drug is an integrated concept, including safety and efficacy, together with attributes such as quality of life, cancer-related symptoms and cost. It is a quantifiable concrete value that can reflect the personalized characteristics of the drug to meet the different preferences of patients. The skyrocketing price of new anti-tumour drugs (especially targeted therapy and immunotherapy drugs), combined with the high burden of cancer, has resulted in an urgent need to assess their value *versus* their cost. The American Society of Clinical Oncology (ASCO) and the European Society for Medical Oncology (ESMO) have developed and updated their conceptual frameworks to assess the benefit of new cancer therapies: the ASCO Value Framework (ASCO-VF) and the ESMO-Magnitude of Clinical Benefit Scale (ESMO-MCBS) (Cherny et al., 2015; Schnipper et al., 2015; Schnipper et al., 2016; Cherny et al., 2017). Both value frameworks aim to quantify the magnitude of value and reasonably assess affordable high-quality therapies for various cancer disease states (Kantarjian et al., 2013). Studies have shown that only one-third of positive trials meet the threshold for meaningful clinical benefit, and not all PD-1/PD-L1 inhibitors meet the threshold in the treatment of cancers (Del Paggio et al., 2017; Jiang et al., 2020).

Considering the inconsistencies in the evidence for PD-1/PD-L1 inhibitors in EC, GC and CRC and the challenge of increasing the tumor burden due to the skyrocketing price of new anti-tumor drugs, we carried out this study to quantify the value of PD-1/PD-L1 inhibitors in the treatment of EC, GC and CRC with ASCO-VF and ESMO-MCBS and to analysis the association between the value and cost of PD-1/PD-L1 inhibitors.

## Methods

### Selection of randomized controlled trials

We systematically searched eight databases, including Cochrane Library, PubMed, Embase, Web of Science (WOS), China National Knowledge Infrastructure (CNKI), Wanfang Data, Chongqing VIP (CQVIP), and Chinese BioMedical Literature Database (CBM), with the search terms “PD-1”, “PD-L1”, “gastric”, “colorectal”, “oesophageal” and “randomized controlled trial” to identify RCTs published from inception to 1 July 2022. The search strategy was preformulated by the research team and finally implemented by a team member (SL Ou). Furthermore, the reference lists of relevant systematic reviews were reviewed, and ClinicalTrials.gov was also checked to avoid omissions. Duplicate studies were removed by Endnote X9. The search strategy is detailed in Supplementary Table S1.

Studies were included that met the following criteria: 1) population: patients with EC, GC, gastroesophageal junction cancer (GEJC) and CRC; 2) intervention: PD-1/PD-L1 inhibitor monotherapy or in combination with chemotherapy (CT); 3) control: placebo or CT; 4) outcomes: hazard ratio (HR) of overall survival (OS), progression-free survival (PFS) or disease-free survival (DFS), grade 1–2 adverse events (AEs) and grade 3–4 AEs, quality of life (QoL); 5) study: Phase 2/3 RCT. Studies were excluded following exclusion criteria: 1) studies did not report survival curves or the rates of grade 1–2 AEs and grade 3–4 AEs; 2) non-Chinese or English literature.

### Framework

The advanced disease and adjuvant or neoadjuvant therapy settings forms of ASCO-VF version 2 and ESMO-MCBS version 1.1 were used to assess the value scores (Schnipper et al., 2016; Cherny et al., 2017). ASCO-VF is designed for only in phase II or III RCT, including clinical benefit, toxicity and bonus points. The net health benefit (NHB) score is obtained by the final sum of the three module scores. The clinical benefit score is subtracted HR value the survival outcome indicator from 1, multiply by 100 points and then multiply by the weight (OS weighted 1, PFS weighted 0.8, ORR weighted 0.7). The toxicity score is the percentage difference between the total toxicity points of the intervention regimen and the control regimen multiply by 20 points. If the intervention regimen is more toxic than the control regimen, the toxicity score is subtracted from the clinical benefit score. If the toxicity of the intervention regimen was lower than the control regimen, the toxicity score is added to the clinical benefit score. Bonus points include 20 points for long-term survival (OS weighted 1, PFS weighted 0.8), 10 points for improvement in cancer-related symptoms, 10 points for quality of life, and percentage improvement in treatment-free interval multiply 20 points.

The ESMO-MCBS framework is designed for use only in positive trials, including clinical benefit, toxicity/quality of life. The clinical benefit grade is based on the lower limit of the 95% confidence interval (*CI)* of HR of survival outcome associated with a particular grade in a prespecified manner (e.g., grade 4 for control regimen with median OS < 12 months, HR ≤ 0.65 and OS gain ≥3 months). Upgraded 1 level if improved quality of life or/and less specific 3–4 AEs are shown.

Finally, the net health benefit (NHB) scores of ASCO-VF are continuous data; ESMO-MCBS grades are distributed as 5, 4, 3, 2 or 1 for advanced disease setting and as A, B, or C for adjuvant or neoadjuvant therapy setting. ASCO-VF does not clearly define what score is considered the “meaningful value threshold”, whereas ESMO-MCBS defines “meaningful clinical benefit” as a grade of 5, 4, A or B.

### Data extraction and scoring

Two authors (SL Ou and JL) independently screened the titles and abstracts and full texts of eligible studies and used a standardized extraction form to extract the data. The extracted contents included the study name, phase, sample size, type of cancer, PD-1/PD-L1 inhibitors used, dosage regimen, follow-up time and outcomes. ASCO-VF scores and ESMO-MCBS grades were also independently evaluated by two authors (SL Ou and XL Qin). Any discrepancies were adjudicated by a third author (HW) to establish the final score or grade.

To assess the monthly cost of all anti-tumor drugs in the intervention and control groups of the included RCTs, we used the price of the branded name and generic drugs (often generic) from the Hospital Information System (HIS), which derived from the lowest wholesale pricing of the centralized procurement and drug price supervision platform of Sichuan Province and represented the actual purchase price of drugs in public medical institutions of the inter-provincial alliance. The monthly cost was calculated according to the dosage schedule in the included RCTs for a patient weighing 60 kg with a body surface area of 1.70 m^2^. We reported the incremental monthly cost as the difference between the intervention and control groups. If the control group was placebo or best supportive care, the cost was set at zero. The most expensive one was recorded when the control group had multiple therapeutic regimens. The monthly cost of the therapeutic regimen was calculated over an average period of 30 days. Therapeutic regimens not available in China were not counted.

### Statistical analysis

All data were collected using a standardized extraction form in an Excel file. Statistical analysis was performed with IBM SPSS (version 25.0). Continuous data were plotted to assess the normality of the underlying distribution. Comparisons between study groups were made using *Student’s* t-test or the *Wilcoxon signed-ranked* test, as appropriate. We generated a receiver operating characteristic (ROC) curve to assess the predictive value of the ASCO-VF score in relation to the threshold of the ESMO-MCBS grade and evaluate the consistency of the two value frameworks. We used scatterplots and Pearson’s or Spearman’s correlation to show the association between incremental monthly cost and ASCO-VF scores or ESMO-MCBS grades. All analyses were deemed significant if *p* < 0.05.

## Results

### Study selection and characteristics

**FIGURE 1:**
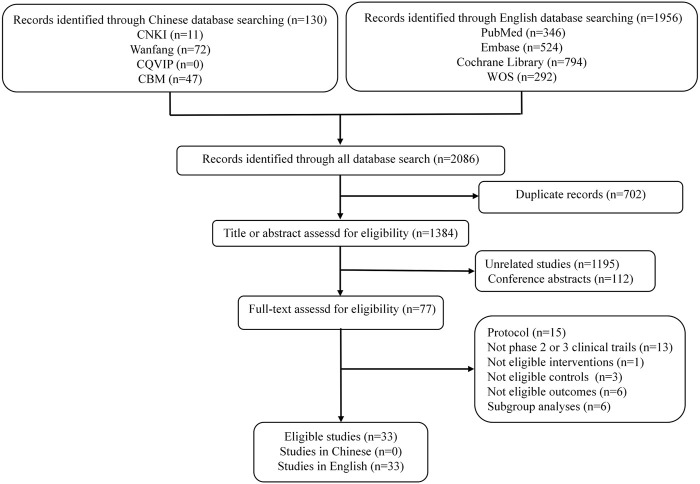
Flow diagram of study selection.

We identified 2086 records through initial retrieval. Ultimately, 33 studies reporting 23 RCTs published in English were considered eligible for this study (Kang et al., 2017; Bang et al., 2018; Shitara et al., 2018; Eng et al., 2019; Kato et al., 2019; Chen E. X. et al., 2020; André et al., 2020; Chen L. T. et al., 2020; Huang et al., 2020; Kojima et al., 2020; Shitara et al., 2020; Andre et al., 2021; Van Cutsem et al., 2021a; Boku et al., 2021; Van Cutsem et al., 2021b; Janjigian et al., 2021; Kelly et al., 2021; Luo et al., 2021; Moehler et al., 2021; Sun et al., 2021; Adenis et al., 2022; Antoniotti et al., 2022; Diaz et al., 2022; Doki et al., 2022; Fuchs et al., 2022; Kang et al., 2022; Lu et al., 2022; Mettu et al., 2022; Okada et al., 2022; Park et al., 2022; Shitara et al., 2022; Wang et al., 2022; Xu et al., 2022) (Figure 1). Of these, two (8.7%) RCTs were conducted in the setting of adjuvant therapy, while the others (91.3%) were conducted in the setting of advanced disease. Ten (43.48%) RCTs involved treatments for EC, five (21.74%) involved treatments for CRC, and eight (34.78%) involved treatments for GC/GEJC. Four (17.4%) RCTs had three arms, and the others (82.6%) had two arms. The median sample size was 493 (*IQR* 307–724), and all included studies were supported by pharmaceutical companies. More characteristics are presented in Table 1.

**TABLE 1:** Characteristics of the included studies.

Registry number	Year	Study code	Phase	Disease type	Setting	Line	Intervention arm	Control arm	PD-L1 expression level	Sample size	Follow- up time m)	Industry sponsorship	Outcomes
NCT02520453 Park et al. (2022)	2022	—	Ⅱ	EC (squamous carcinoma)	Adjuvant	—	Durvalumab	Placebo	—	86 (45/41)	38.7	Yes	OS, DFS, AEs
NCT02743494 Kelly et al. (2021)	2021	Checkmate 577	III	EC/GEJC	Adjuvant	—	Nivolumab	Placebo	—	894 (532/262)	24.4	Yes	DFS, AEs
NCT02873195 Mettu et al. (2022)	2022	BACCI	Ⅱ	CRC	Advanced	2	Atezolizumab + Bevacizumab + Capecitabine	Placebob + Bevacizumab + Capecitabine	—	133 (86/47)	20.9	Yes	OS, PFS, ORR, AEs
NCT02563002 André et al. (2020); Andre et al. (2021); Diaz et al. (2022)	2020	KEYNOTE-177	III	CRC	Microsatellite instability–high advanced	1	Pembrolizumab	Oxaliplatin + Leucovorin+5-fluoropyrimidine + Bevacizumab or Cetuximab	—	307 (153/154)	44.5	Yes	PFS, ORR, AEs
NCT02788279 Eng et al. (2019)	2019	IMblaze370	III	CRC	Advanced	3	Atezolizumab + Cobimetinib Atezolizumab	Regorafenib	—	363 (183/90/90)	7.3	Yes	OS, PFS, ORR, AEs
NCT03721653 Antoniotti et al. (2022)	2022	Atezo TRIBE	Ⅱ	CRC (adenocarcinoma)	Advanced	1	Atezolizumab + Bevacizuma + Irinotecan + Oxaliplatin + Leucovorin+5-fluoropyrimidine	Bevacizuma + Irinotecan + Oxaliplatin + Leucovorin+5-fluoropyrimidine	—	218 (145/73)	19.9	Yes	PFS, ORR, AEs
NCT02870920 Chen et al. (2020a)	2020	—	Ⅱ	CRC (adenocarcinoma)	Advanced	≥3	Durvalumab + Tremelimumab + Best supportive care	Best supportive care	—	180 (119/61)	15.2	Yes	OS, PFS, AEs
NCT02564263 Kojima et al. (2020); Adenis et al. (2022)	2020	KEYNOTE-181	III	EC	Advanced	2	Pembrolizumab	Paclitaxel or Docetaxel or Irinotecan	—	628 (314/314)	11.1	Yes	OS, PFS, ORR, AEs
	2021												
NCT03189719 Sun et al. (2021)	2021	KEYNOTE-590	III	EC	Advanced	1	Pembrolizumab+5-fluoropyrimidine + Cisplatin	Placebo+5-fluoropyrimidine + Cisplatin	—	749 (373/376)	22.6	Yes	OS, PFS, ORR, AEs
NCT03116152 Xu et al. (2022)	2022	ORIENT-2	Ⅱ	EC (squamous carcinoma)	Advanced	2	Sintilimab	Paclitaxel or Irinotecan	—	190 (95/95)	7.2	Yes	OS, PFS, ORR, AEs
NCT03143153 Doki et al. (2022)	2022	CheckMate 648	III	EC (squamous carcinoma)	Advanced	1	Nivolumab+5-fluoropyrimidine + Cisplatin Nivolumab + Ipilimumab	5-fluoropyrimidine + Cisplatin	—	970 (321/325/324)	13	Yes	OS, PFS, ORR, AEs
NCT03748134 Lu et al. (2022)	2022	ORIENT-15	III	EC (squamous carcinoma)	Advanced	1	Sintilimab+(Cisplatin + Paclitaxel) or (5-fluoropyrimidine + Cisplatin)	Placebo+(Cisplatin + Paclitaxel) or (5-fluoropyrimidine + Cisplatin)	—	659 (327/332)	16.9	Yes	OS, PFS, ORR, AEs
NCT03829969 Wang et al. (2022)	2022	JUPITER-06	III	EC (squamous carcinoma)	Advanced	1	Toripalimab + Cisplatin + Paclitaxel	Cisplatin + Paclitaxel	—	514 (257/257)	7.1	Yes	OS, PFS, ORR, AEs
NCT03691090 Luo et al. (2021)	2021	ESCORT-1	III	EC (squamous carcinoma)	Advanced	1	Camrelizumab + Cisplatin + Paclitaxel	Placebo + Cisplatin + Paclitaxel	—	596 (298/298)	10.8	Yes	OS, PFS, ORR, AEs
NCT03099382 Huang et al. (2020)	2020	ESCORT	III	EC (squamous carcinoma)	Advanced	2	Camrelizumab	Docetaxel or Irinotecan	—	457 (229/228)	8.3	Yes	OS, PFS, ORR, AEs
NCT02569242 Kato et al. (2019); Okada et al. (2022)	2019	ATTRACTION-3	III	EC (squamous carcinoma)	Advanced	2	Nivolumab	Paclitaxel or Docetaxel	—	419 (210/209)	36	Yes	OS, PFS, ORR, AEs
NCT02872116 Janjigian et al. (2021); Shitara et al. (2022)	2021	CheckMate 649	III	GC/EC/GEJC (adenocarcinoma)	Advanced	1	Nivolumab+(Capecitabine + Oxaliplatin) or (Oxaliplatin + Leucovorin+5-fluoropyrimidine) Nivolumab + Ipilimumab	(Capecitabine + Oxaliplatin) or (Oxaliplatin + Leucovorin+5-fluoropyrimidine)	—	2031 (789/792/450)	24	Yes	OS, PFS, ORR, AEs
NCT02746796 Kang et al. (2022)	2022	ATTRACTION-4	III	GC/GEJC	HER2-negative advanced	1	Nivolumab + Capecitabine + Oxaliplatin	Placebo + Capecitabine + Oxaliplatin	—	724 (362/362)	26.5	Yes	OS, PFS, ORR, AEs
NCT02625623 Bang et al. (2018)	2018	JAVELIN Gastric 300	III	GC/GEJC	Advanced	3	Avelumab	Paclitaxel or Irinotecan	—	371 (185/186)	10.6	Yes	OS, PFS, ORR, AEs
NCT02267343 Kang et al. (2017); Chen et al. (2020b); Boku et al. (2021)	2017	ATTRACTION-2	III	GC/GEJC	Advanced	3	Nivolumab	Placebo	—	493 (330/163)	36	Yes	OS, PFS, ORR, AEs
NCT02494583 Shitara et al. (2020); Van Cutsem et al. (2021b)	2020	KEYNOTE-062	III	GC/GEJC (adenocarcinoma)	Advanced	1	Pembrolizumab Pembrolizumab + Cisplatin or Capecitabine	Placebo + Cisplatin or Capecitabine	PD-L1 CPS≥1	763 (256/257/250)	29.4	Yes	OS, PFS, ORR, AEs
NCT02625610 Moehler et al. (2021)	2020	JAVELIN Gastric 100	III	GC/GEJC (adenocarcinoma)	Advanced	1	Avelumab	Oxaliplatin + Leucovorin+5-fluoropyrimidine	—	499 (249/250)	24	Yes	OS, PFS, ORR, AEs
NCT02370498 Shitara et al. (2018); Van Cutsem et al. (2021a); Fuchs et al. (2022)	2018	KEYNOTE-061	III	GC/GEJC (adenocarcinoma)	Advanced	2	Pembrolizumab	Paclitaxel	PD-L1 CPS≥1	395 (196/199)	52	Yes	OS, PFS, ORR, AEs

Note: EC, oesophageal cancer; GC, gastric cancer; GEJC, gastroesophageal junction cancer; CPS, combined positive score; OS, overall survival; PFS, progression-free survival; ORR, objective response rate; DFS, disease-free survival; AEs, adverse events; /, not reported.

### Value scores/grades

For the adjuvant therapy setting, durvalumab showed a negative value even compared with placebo, with an ASCO-VF score of −18.7. The application of ESMO-MCBS for nivolumab *versus* placebo resulted in a grade of A, which met the meaningful value threshold. For advanced diseases, all 25 therapeutic regimens met the evaluation criteria of ASCO-VF. The scores were normally distributed, ranging from −12.5 to 69. Since ASCO-VF has no clearly defined threshold for the meaningful value threshold, we used the mean score of 26.5 (95% *CI* 18.4–34.6) for subsequent analyses. Therefore, 12 (48%) regimens fell above the threshold, and 13 (52%) regimens fell below the threshold. The mean score of positive therapeutic regimens was 37.2 (95% *CI* 27.6–49.2), and the mean score of negative therapeutic regimens was 12.8 (95% *CI* 3.4–22.2). The value score of positive therapeutic regimens was significantly higher than that of negative therapeutic regimens (*p* < 0.001, *Student’s* t-test). Fourteen positive therapeutic regimens met the evaluation criteria of ESMO-MCBS. Six (42.9%) of the regimens met the ESMO-MCBS benefit threshold grade, and eight (57.1%) of the regimens did not meet the ESMO-MCBS benefit threshold grade (Table 2).

**TABLE 2:** Clinical benefit according to ASCO-VF and ESMO-MCBS.

Registry number	Intervention arm	Primary outcome	Primary outcome HR (95% *CI*)	ASCO-VF				ESMO-MCBS			Monthly incremental cost (¥)
				Clinical benefit score	Toxicity score	Bonus points	NHB	Clinical benefit grade	Quality of life/Grade 3–4 toxicities	ESMO-MCBS	
NCT02520453 Park et al. (2022)	Durvalumab	OS	1.08 (0.52–2.24)	−8	−10.7	0	−18.7	NA	NA	NA	51756.92
NCT02743494 Kelly et al. (2021)	Nivolumab	DFS	0.69 (0.56–0.86)	31	−10	0	21	A	0	A	49471.59
NCT02873195 Mettu et al. (2022)	Atezolizumab + CT	OS	0.96 (0.63–1.45)	4	−2	0	2	NA	NA	NA	46857.14
NCT02563002 André et al. (2020); Andre et al. (2021); Diaz et al. (2022)	Pembrolizumab	PFS	0.59 (0.45–0.79)	32.8	13.6	20	66.4	3	1	4	24579.4
NCT02788279 Eng et al. (2019)	Atezolizumab + Cobimetinib	OS	1.00 (0.73–1.38)	0	−0.4	0	−0.4	NA	NA	NA	—
	Atezolizumab	OS	1.19 (0.83–1.71)	−19	6.5	0	−12.5	NA	NA	NA	26159.54
NCT03721653 Antoniotti et al. (2022)	Atezolizumab + CT	PFS	0.69 (0.56–0.85)	24.8	−5.3	0	19.5	2	0	2	70285.71
NCT02870920 Chen et al. (2020a)	Durvalumab + Tremelimumab	OS	0.72 (0.54–0.97)	28	−5.9	0	22.1	3	0	3	—
NCT02564263 Kojima et al. (2020); Adenis et al. (2022)	Pembrolizumab	OS	0.89 (0.75–1.05)	11	20	0	31	NA	NA	NA	39537.14
NCT03189719 (Sun et al. (2021)	Pembrolizumab + CT	OS	0.73 (0.62–0.86)	27	−1.9	20	45.1	4	0	4	51194.29
NCT03116152 Xu et al. (2022)	Sintilimab	OS	0.70 (0.50–0.97)	30	7.5	0	37.5	1	1	2	−8571.43
NCT03143153 Doki et al. (2022)	Nivolumab + CT	OS	0.74 (0.58–0.96)	26	−3.1	0	22.9	3	0	3	49471.59
	Nivolumab + Ipilimumab	OS	0.78 (0.62–0.98)	22	9	0	31	3	0	3	79478.89
NCT03748134 Lu et al. (2022)	Sintilimab + CT	OS	0.63 (0.51–0.78)	37	−1.8	0	35.2	3	0	3	3085.71
NCT03829969 Wang et al. (2022)	Toripalimab + CT	OS	0.58 (0.43–0.78)	42	−3.2	0	38.8	4	0	4	2732.8
NCT03691090 Luo et al. (2021)	Camrelizumab + CT	OS	0.70 (0.56–0.88)	30	−1.9	20	48.1	3	1	4	4182.86
NCT03099382 Huang et al. (2020)	Camrelizumab	OS	0.71 (0.57–0.87)	29	20	20	69	3	1	4	−5382.86
NCT02569242 Kato et al. (2019); Okada et al. (2022)	Nivolumab	OS	0.79 (0.64–0.97)	21	17.5	10	48.5	3	1	4	47989.56
NCT02872116 Janjigian et al. (2021); Shitara et al. (2022)	Nivolumab + CT	OS	0.79 (0.71–0.88)	21	−1.7	0	19.3	2	0	2	49471.59
	Nivolumab + Ipilimumab	OS	0.91 (0.77–1.07)	9	3.2	0	12.2	NA	NA	NA	173104.97
NCT02746796 Kang et al. (2022)	Nivolumab + CT	OS	0.90 (0.75–1.08)	10	−1.3	0	8.7	NA	NA	NA	52638.51
NCT02625623 Bang et al. (2018)	Avelumab	OS	1.11 (0.90–1.40)	−11	15.6	0	4.6	NA	NA	NA	—
NCT02267343 Kang et al. (2017); Chen et al. (2020b); Boku et al. (2021)	Nivolumab	OS	0.62 (0.50–0.75)	38	−20	0	18	1	0	1	39478.89
NCT02494583 Shitara et al. (2020); Van Cutsem et al. (2021b)	Pembrolizumab	OS	0.91 (0.69–1.18)	9	20	0	29	NA	NA	NA	51194.29
	Pembrolizumab + CT	OS	0.85 (0.70–1.03)	15	0.2	0	15.2	NA	NA	NA	50511.43
NCT02625610 Moehler et al. (2021)	Avelumab	OS	0.91 (0.74–1.11)	9	20	0	29	NA	NA	NA	—
NCT02370498 Shitara et al. (2018); Van Cutsem et al. (2021a); Fuchs et al. (2022)	Pembrolizumab	OS	0.81 (0.66–1.00)	19	2.8	0	21.8	NA	NA	NA	50113.64

Note: CT, chemotherapy; OS, overall survival; PFS, progression-free survival; DFS, disease-free survival; NHB, net health benefit; NA, not applicable; /: not available in China.

The ROC curve was used to forecast the meaningful value threshold of ASCO-VF to meet the ESMO-MCBS in advanced disease. The threshold score was 38.2, which was close to that in our previous study (Jiang et al., 2020). Excitingly, the area under the curve was 1.0 (*p* = 0.002), suggesting exactly the same predictive value. Based on this result, ASCO-VF scores and ESMO-MCBS grades showed that pembrolizumab met the meaningful value threshold in the first-line treatment of EC and microsatellite instability–high CRC. Toripalimab and camrelizumab met meaningful value threshold in the first-line treatment of squamous cell EC, and nivolumab and camrelizumab met meaningful value threshold in second-line treatment. PD-1/PD-L1 inhibitors did not meet valuable threshold in GC/GEJC.

### Correlation between value scores/grades and cost

**FIGURE 2:**
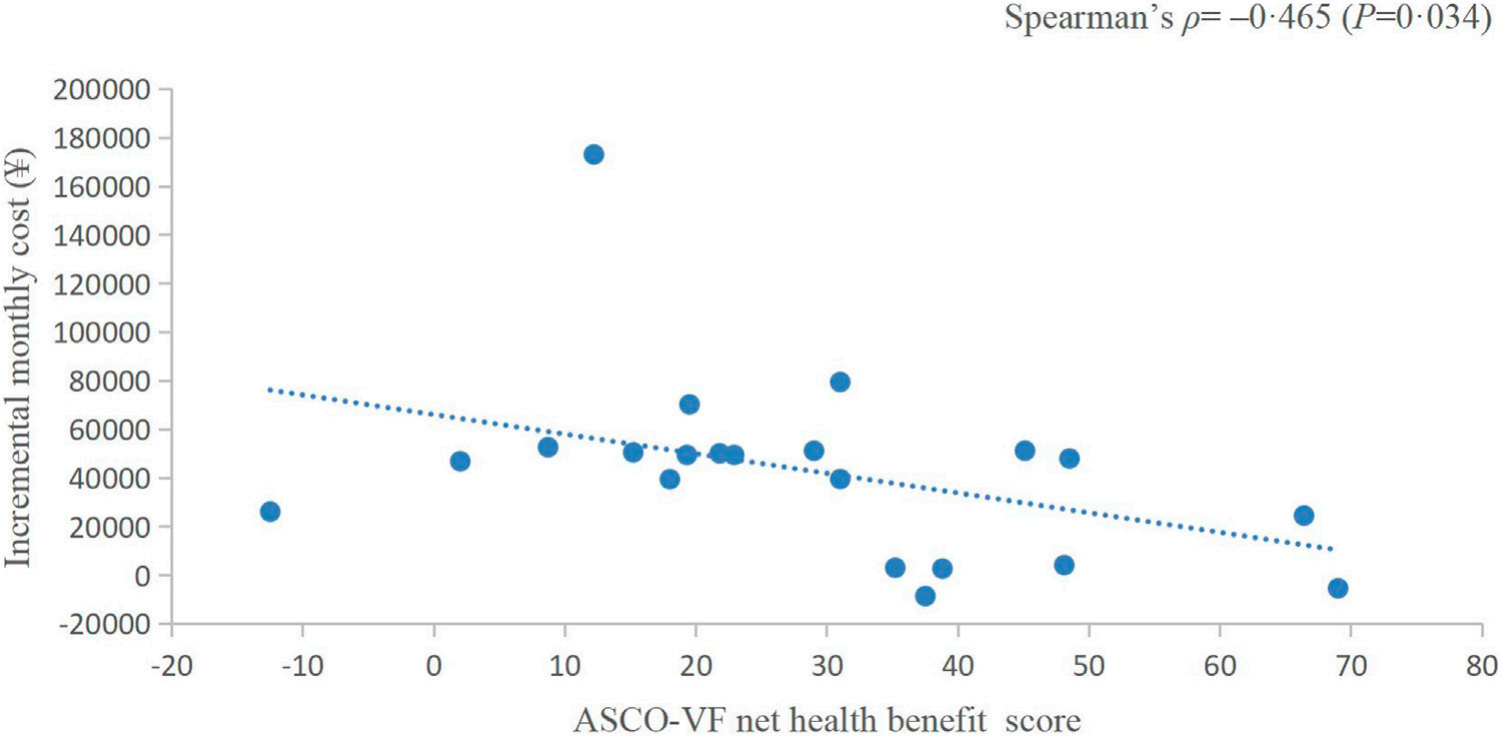
Scatterplot of the correlation between ASCO-VF net health benefit scores and incremental monthly cost.

**FIGURE 3:**
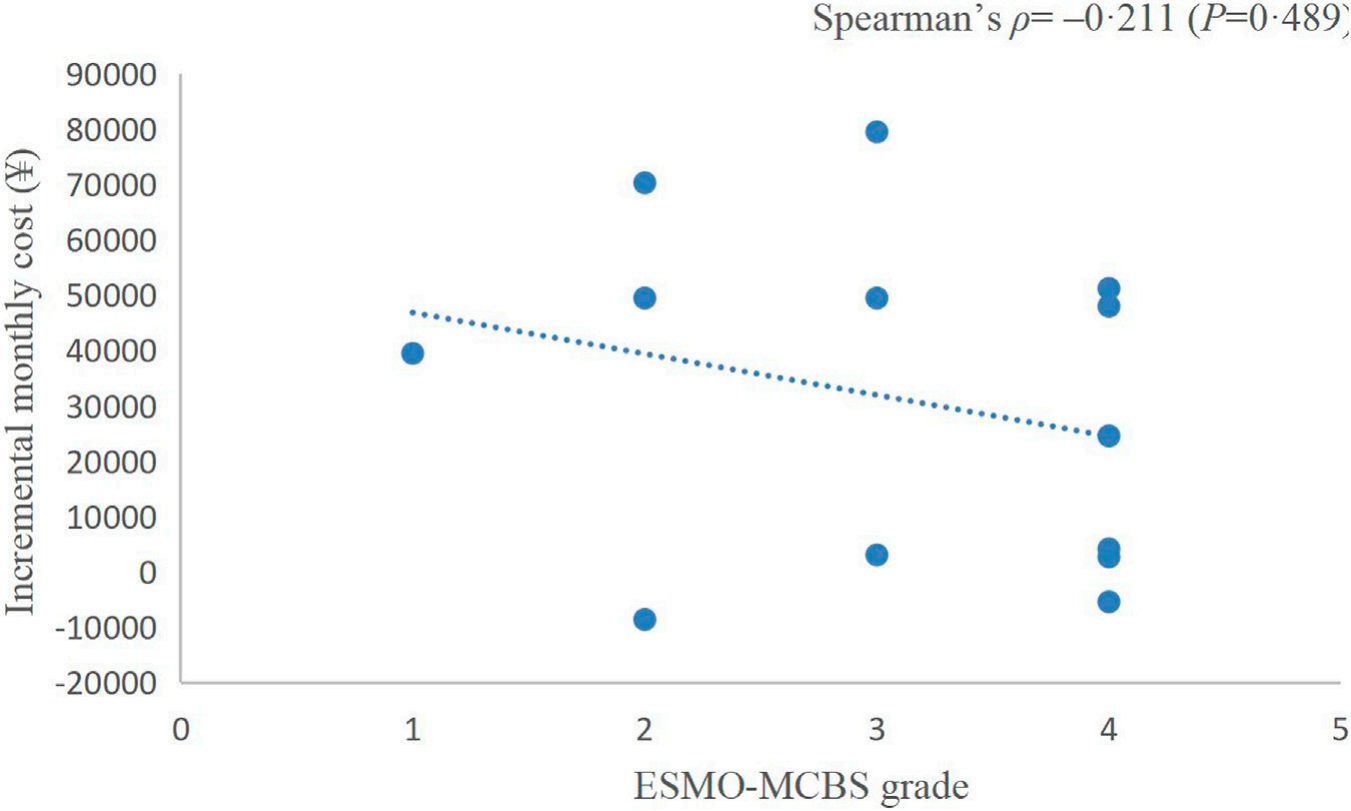
Scatterplot of correlation between ESMO-MCBS grades and incremental monthly cost.

The incremental monthly cost data of RCTs assessed by ASCO-VF were not normally distributed, thus, we analysed the correlation between value scores/grades and incremental monthly cost with Spearman’s correlation. The incremental monthly cost and ASCO-VF scores were negatively correlated (Spearman’s *ρ* = −0.465, *p* = 0.034, Figure 2). For ESMO-MCBS grades, the incremental monthly cost and value grades also showed a negative correlation (Spearman’s *ρ* = −0.211, *p* = 0.489, Figure 3).

## Discussion

### Summary of results

The rising price of new anticancer drugs has led to public criticism of the pricing policies of manufacturers (Kantarjian et al., 2013). Coupled with the high burden of cancer, value assessment of new anti-tumor drugs has become an urgent need (Bach and Pearson, 2015). In this study, we assessed the value of PD-1/PD-L1 inhibitors in EC, GC and CRC using ASCO-VF and ESMO-MCBS. We found that only a few treatment regimens showed clinical value in EC and CRC. The association between ASCO-VF and ESMO-MCBS in this study was very well, and the value score/grade was negatively correlated with the incremental monthly cost.

Adjuvant chemotherapy after surgery is generally required for resectable locally advanced EC or GEJC. However, no treatment regimen has been shown to be effective, and the standard of care is best supportive care (Stahl et al., 2013; Ajani et al., 2019). In our study, nivolumab met valuable threshold in resectable locally advanced EC/GEJC (Kelly et al., 2021), which provides a new reference for clinical treatment and a new direction for clinical trials.

In regard to advanced diseases, 14 positive therapeutic regimens of 13 trials were assessed with both ASCO-VF and ESMO-MCBS, and 11 negative therapeutic regimens of 9 trials were assessed with only ASCO-VF. The NHB scores of positive trials were significantly higher than those of negative trials, and all negative trial scores were below the threshold predicted by the ROC curve. Considering that none of the 11 negative therapeutic regimens showed an improvement in QoL, we may conclude that a treatment is of no value when survival outcomes are not significantly increased while QoL is not improved, which is consistent with the use of ESMO-MCBS for non-inferiority (equivalence) studies (Cherny et al., 2015; Cherny et al., 2017). In GC/GEJC, none of the therapeutic regimens achieved the threshold value score or grade even when the PD-L1 combined positive score (CPS) was ≥1. PD-L1 inhibitor monotherapy or in combination with CT did not reach the threshold in CRC, but the PD-1 inhibitor pembrolizumab showed clinical value with an improvement in efficacy, toxicity and QoL as first-line therapy for microsatellite instability–high CRC (André et al., 2020; Andre et al., 2021; Diaz et al., 2022). In EC, pembrolizumab, toripalimab or camrelizumab in combination with CT showed clinical value in first-line treatment (Luo et al., 2021; Sun et al., 2021; Wang et al., 2022), and nivolumab and camrelizumab monotherapy showed value in second-line treatment (Kato et al., 2019; Huang et al., 2020; Okada et al., 2022). Although significant differences in survival outcomes have been at the forefront of drug approval and clinical decisions for many years, various stakeholders are increasingly focusing on the value (Vivot et al., 2017). In our study, we found that 8 of 14 positive therapeutic regimens did not meet the threshold value (Kang et al., 2017; Chen E. X. et al., 2020; Janjigian et al., 2021; Antoniotti et al., 2022; Doki et al., 2022; Lu et al., 2022; Xu et al., 2022), which suggests that the majority of positive interventions improved overall survival while compromising QoL or increasing the risk of toxicity. Therapeutic decisions should not be made solely on the *p* < 0.05 of survival indicators, and the clinical value of therapeutic regimens should be considered comprehensively.

Traditionally, we assume that the high price of new drugs is due to the need to support research; however, an analysis of transformative drugs shows that the main source of drug innovation is government-funded academic research (Kesselheim et al., 2015). As the payer of medical activities, the price paid by patients for drugs should have a positive relationship with the value created. In recent years, a series of studies have shown that there is no statistically significant association between the value and prices of anticancer drugs (Vivot et al., 2017; Jiang et al., 2020; Vokinger et al., 2020). Interestingly, in this study, we found a negative correlation between the prices of PD-1/PD-L1 inhibitors and their value. This negative correlation between prices and the ASCO-VF value score was even statistically significant (Spearman’s *ρ* = −0.465, *p* = 0.034), resulting in an urgent demand for value-based pricing. Camrelizumab and toripalimab showed clinical value in EC and have relatively low prices in the Chinese market, so their value may be more worthy of payment, which was also consistent with the results of China’s national price negotiations (Zhang et al., 2022).

### Implications

This study has some implications. Firstly, this study shows no clinical value for PD-1/PD-L1 inhibitors in GC/GEJC, which suggests that subsequent clinical trials on the treatment of GC/GEJC with PD-1/PD-L1 inhibitors should fully follow the current evidence. Secondly, the prices of PD-1/PD-L1 inhibitors are not aligned with their value. Price negotiation for higher-priced PD-1/PD-L1 inhibitors should be prioritized to improve patient access to beneficial drugs, thereby contributing to patient-centred cancer treatment goals. Thirdly, all therapeutic regimens with improved QoL showed clinical value (Kato et al., 2019; Huang et al., 2020; Luo et al., 2021; Sun et al., 2021), suggesting that clinical trials and clinical treatment strategies should pay more attention to QoL.

### Limitations

We comprehensively assessed the value of PD-1/PD-L1 inhibitors in oesophageal and gastrointestinal cancer with ASCO-VF and ESMO-MCBS, and we acknowledged some limitations. Firstly, the number of RCTs included in this study was small, and there were only 14 therapeutic regimens that met both the ASCO-VF and ESMO-MCBS criteria. Although the consistency of the two value frameworks in this study was perfect, the conclusion may exist the risk of bias due to the influence of the small sample size. Secondly, as ASCO-VF did not define toxicity scores for subgroup analyses, they could not be evaluated in the subgroup analyses. Therefore, the subgroup results of PD-L1 expression and microsatellite stability level were partially incomplete. Thirdly, we used the pricing system of public hospitals and centralized procurement and drug price supervision platforms of Sichuan province in China, so the results of the correlation between the value scores/grades and cost do not necessarily apply to countries outside of China. Finally, we only considered drug costs when calculating monthly increments, without taking into account the patients and their spouses or other important people due to absence, emergency treatment, hospitalization and medical expenses. In fact, because these costs are not easy to obtain directly, value frameworks consider only the cost of drugs as a rough estimate of the cost of treatment.

## Conclusion

ASCO-VF and ESMO-MCBS could identify therapeutic regimens with clinical value. The incremental monthly cost for PD-1/PD-L1 inhibitors was not proportional to their value. PD-1/PD-L1 inhibitors did not meet valuable threshold in GC/GEJC. Pembrolizumab met the valuable threshold in advanced microsatellite instability–high CRC. The value of camrelizumab and toripalimab may be more worth paying in EC.

## Data Availability

The original contributions presented in the study are included in the article/Supplementary Material, further inquiries can be directed to the corresponding author.
