# Supplementary material for: Value assessment of PD-1/PD-L1 inhibitors in the treatment of oesophageal and gastrointestinal cancers
Source: Front Pharmacol. 2023 Apr 21;14:1106961. doi: 10.3389/fphar.2023.1106961 (PMC10160363; doi:10.3389/fphar.2023.1106961)
Supplement: Supplementary file 1 [file Table1.DOCX]

*Supplementary Table S1：P1-4*

**Table S1. Searching Strategy**

| **Database** | **Search Strategy** | **Results** |
| --- | --- | --- |
| PUBMED | #1 gastrointestinal neoplasms[MeSH Terms]#2 esophageal[Title/Abstract] OR gastric[Title/Abstract] OR gastroesophageal junction[Title/Abstract] OR colorectal[Title/Abstract]#3 tumor[Title/Abstract] OR cancer[Title/Abstract] OR carcinoma[Title/Abstract]#4 #2 AND #3#5 #1 OR #4#6 pembrolizumab[Supplementary Concept] OR pembrolizumab[Title/Abstract] OR SCH-900475[Title/Abstract] OR keytruda[Title/Abstract] OR MK-3475[Title/Abstract] OR lambrolizumab[Title/Abstract]#7 nivolumab[Mesh] OR nivolumab[Title/Abstract] OR opdivo[Title/Abstract] OR ONO 4538[Title/Abstract] OR ONO-4538[Title/Abstract] OR ONO4538[Title/Abstract] OR MDX-1106[Title/Abstract] OR MDX 1106[Title/Abstract] OR MDX1106 OR BMS-936558[Title/Abstract] OR BMS 936558 OR BMS936558#8 toripalimab[Supplementary Concept] OR toripalimab[Title/Abstract]#9 sintilimab[Supplementary Concept] OR sintilimab[Title/Abstract] OR IBI 308[Title/Abstract] OR IBI308[Title/Abstract] OR IBI-308[Title/Abstract]#10 camrelizumab[Supplementary Concept] OR camrelizumab[Title/Abstract] OR SHR-1210[Title/Abstract] OR SHR 1210 #11 durvalumab[Supplementary Concept] OR durvalumab[Title/Abstract] OR MEDI4736[Title/Abstract] OR MEDI-4736 OR imfinzi[Title/Abstract]  #12 tislelizumab[Supplementary Concept] OR tislelizumab[Title/Abstract]  #13 atezolizumab[Supplementary Concept] OR atezolizumab[Title/Abstract] OR anti-PDL1[Title/Abstract] OR immunoglobulin G1[Title/Abstract] OR MPDL3280A[Title/Abstract] OR MPDL-3280A[Title/Abstract] OR tecentriq[Title/Abstract] OR RG7446[Title/Abstract] OR RG-7446[Title/Abstract]  #14 avelumab[Supplementary Concept] OR avelumab[Title/Abstract] OR MSB-0010682[Title/Abstract] OR MSB0010682[Title/Abstract] OR bavencio[Title/Abstract] OR MSB0010718C[Title/Abstract] OR MSB-0010718C[Title/Abstract]  #15 immune checkpoint inhibitors[Mesh] OR immune checkpoint inhibitors[Title/Abstract] OR PD-L1 inhibitors[Title/Abstract] OR programmed death-Ligand-1 inhibitors[Title/Abstract] OR PD-1 inhibitors[Title/Abstract] OR programmed cell death protein-1 inhibitors[Title/Abstract]  #16 #6 OR #7 OR #8 OR #9 OR #10 OR #11 OR #12 OR #13 OR #14 OR #15  #17 ("randomized controlled trial"[Publication Type] OR "controlled clinical trial"[Publication Type] OR "randomized"[Title/Abstract] OR "placebo"[Title/Abstract] OR "clinical trials as topic"[MeSH Terms:noexp] OR "randomly"[Title/Abstract] OR "trial"[Title]) NOT ("animals"[MeSH Terms] NOT "humans"[MeSH Terms])  #18 #5 AND #16 AND #17 | 346 |
| EMBASE | #1 'gastrointestinal neoplasms'/exp  #2 esophageal:ti,ab,kw OR gastric:ti,ab,kw OR gastroesophageal:ti,ab,kw OR colorectal:ti,ab,kw  #3 tumor:ti,ab,kw OR cancer:ti,ab,kw OR carcinoma:ti,ab,kw  #4 #2 AND #3  #5 #1 OR #4  #6 'pembrolizumab'/exp OR pembrolizumab:ti,ab,kw OR 'sch 900475':ti,ab,kw OR keytruda:ti,ab,kw OR 'mk 3475':ti,ab,kw OR lambrolizumab:ti,ab,kw  #7 ''nivolumab'/exp OR nivolumab:ti,ab,kw OR opdivo:ti,ab,kw OR 'ono 4538':ti,ab,kw OR 'mdx 1106':ti,ab,kw OR 'bms 936558':ti,ab,kw  #8 'toripalimab'/exp OR toripalimab:ti,ab,kw  #9 'sintilimab'/exp OR sintilimab:ti,ab,kw OR 'ibi 308':ti,ab,kw  #10 'camrelizumab'/exp OR camrelizumab:ti,ab,kw OR 'shr 1210':ti,ab,kw  #11 'tislelizumab'/exp OR tislelizumab:ti,ab,kw  #12 'durvalumab'/exp OR durvalumab:ti,ab,kw OR 'medi 4736':ti,ab,kw OR imfinzi:ti,ab,kw  #13 'atezolizumab'/exp OR atezolizumab:ti,ab,kw OR 'anti pdl1':ti,ab,kw OR 'immunoglobulin g1':ti,ab,kw OR 'mpdl 3280a':ti,ab,kw OR tecentriq:ti,ab,kw OR 'rg 7446':ti,ab,kw  #14 'avelumab'/exp OR avelumab:ti,ab,kw OR 'msb 0010682':ti,ab,kw OR 'msb 0010718c':ti,ab,kw OR bavencio:ti,ab,kw  #15 'immune checkpoint inhibitors'/exp OR 'immune checkpoint inhibitors':ti,ab,kw OR 'pd-l1 inhibitors':ti,ab,kw OR 'programmed death-ligand 1 inhibitors':ti,ab,kw OR 'pd-1 inhibitors':ti,ab,kw OR 'programmed cell death protein 1 inhibitors':ti,ab,kw  #16 #6 OR #7 OR #8 OR #9 OR #10 OR #11 OR #12 OR #13 OR #14 OR #15  #17 #5 AND #16  #18 #17 AND 'controlled clinical trial'/de  #19 #17 AND [randomized controlled trial]/lim  #20 #18 OR #19 | 524 |
| COCHRANE | #1 MeSH descriptor: [gastrointestinal neoplasms] explode all trees#2 (esophageal OR gastric OR gastroesophageal OR colorectal):ti,ab,kw#3 (tumor OR cancer OR carcinoma):ti,ab,kw#4 #2 AND #3#5 #1 OR #4#6 (pembrolizumab):ti,ab,kw OR (SCH-900475):ti,ab,kw OR (Keytruda):ti,ab,kw OR (MK-3475):ti,ab,kw OR (lambrolizumab):ti,ab,kw#7 MeSH descriptor: [nivolumab] explode all trees#8 (nivolumab):ti,ab,kw OR (opdivo):ti,ab,kw OR (ONO-4538):ti,ab,kw OR (MDX-1106):ti,ab,kw OR (BMS-936558):ti,ab,kw#9 (toripalimab):ti,ab,kw#10 (sintilimab):ti,ab,kw OR (IBI-308):ti,ab,kw#11 (camrelizumab):ti,ab,kw OR (SHR-1210):ti,ab,kw#12 (tislelizumab):ti,ab,kw#13 (durvalumab):ti,ab,kw OR (MEDI-4736):ti,ab,kw OR (imfinzi):ti,ab,kw#14 (atezolizumab):ti,ab,kw OR (anti-PDL1):ti,ab,kw OR (immunoglobulin G1):ti,ab,kw OR (MPDL-3280A):ti,ab,kw OR (Tecentriq):ti,ab,kw#15 (avelumab):ti,ab,kw OR (MSB-0010682):ti,ab,kw OR (MSB-0010718C):ti,ab,kw OR bavencio:ti,ab,kw#16 (immune checkpoint inhibitors):ti,ab,kw OR (PD-L1 Inhibitors):ti,ab,kw OR (programmed death-ligand 1 inhibitors):ti,ab,kw OR (PD-1 inhibitors):ti,ab,kw OR (programmed cell death protein 1 inhibitors):ti,ab,kw#17 #6 OR #7 OR #8 OR #9 OR #10 OR #11 OR #12 OR #13 OR #14 OR #15 OR #16#18 #5 AND #17#19 #18 in Trials | 794 |
| WEB of SCIENCE | #1 TS=(neoplasms OR tumor OR cancer OR carcinoma) #2 TS=(gastrointestinal OR esophageal OR gastric OR gastroesophageal OR colorectal)  #3 #1 AND #2  #4 TS=(pembrolizumab OR SCH-900475 OR keytruda OR MK-3475 OR lambrolizumab)  #5 TS=( nivolumab OR opdivo OR ONO 4538 OR ONO-4538 OR ONO4538 OR MDX-1106 OR MDX 1106 OR MDX1106 OR BMS-936558 OR BMS 936558 OR BMS936558)  #6 TS=(toripalimab)  #7 TS=( sintilimab OR IBI 308 OR IBI308 OR IBI-308)  #8 TS=(camrelizumab OR SHR-1210 OR SHR 1210)  #9 TS=(durvalumab OR MEDI4736 OR MEDI-4736 OR imfinzi)  #10 TS=(tislelizumab)  #11 TS=(atezolizumab OR anti-PDL1 OR immunoglobulin G1 OR MPDL3280A OR MPDL-3280A OR tecentriq OR RG7446 OR RG-7446)  #12 TS=(avelumab OR MSB-0010682 OR MSB0010682 OR bavencio OR MSB0010718C OR MSB-0010718C)  #13 TS=(immune Checkpoint Inhibitors OR PD-L1 Inhibitors OR programmed death-ligand 1 inhibitors OR PD-1 inhibitors OR programmed cell death protein 1 inhibitors)  #14 #4 OR #5 OR #6 OR #7 OR #8 OR #9 OR #10 OR #11 OR #12 OR #13  #15 TS=randomized clinical trial  #16 #3 AND #14 AND #15 | 292 |
